# SSR2 overexpression associates with tumorigenesis and metastasis of Hepatocellular Carcinoma through modulating EMT

**DOI:** 10.7150/jca.44788

**Published:** 2020-07-20

**Authors:** Xiaopeng Hong, Hui Luo, Genglong Zhu, Xiaodong Guan, Yingbin Jia, Hailing Yu, Xiufang Lv, Ting Yu, Huimin Lan, Qianqian Zhang, Hanjie Li, Weiming Sun, Xiaofang Huang, Jian Li

**Affiliations:** 1 Department of Hepatobiliary Surgery, The Fifth Affiliated Hospital, Sun Yat-sen University, Zhuhai, Guangdong Province 519000, P. R. China.; 2 Guangdong Provincial Key Laboratory of Biomedical Imaging and Guangdong Provincial Engineering Research Center of Molecular Imaging, The Fifth Affiliated Hospital, Sun Yat-sen University, Zhuhai, Guangdong Province 519000, P. R. China.

**Keywords:** hepatocellular carcinoma, tumorigenesis, SSR2, EMT

## Abstract

**Background:** Hepatocellular carcinoma (HCC) is a common malignancy around the world. The molecular mechanisms underlying HCC tumorigenesis and metastasis are far from clear. Numerous studies have pointed out that signal sequence receptor (SSR) is an endoplasmic reticulum-related protein involved in protein folding and processing of eukaryotic cells. SSR2 is a subunit of SSR protein, but the role of SSR2 in hepatocellular carcinoma is largely unknown and warrants further study.

**Materials and Methods:** Several public databases were data mined to analyze the expression of four subunits of SSR between tumor and its peritumor counterparts. Also, the expression of SSR2 in our own collected tissues from HCC patients were analyzed by IHC and quantitative PCR. Survival analyses were conducted to delineate the prognostic value of SSR2. Clinical data were obtained followed by analysis based on SSR2 expression. Afterwards, cell proliferation, migration and invasion were detected by IncuCyte and trans-well assays, respectively. RNA interference was carried out by transfecting specific siRNA targeting SSR2 into cells using lipo2000. Western blot was applied to validate the knockdown effect and regulation on EMT-related proteins.

**Results:** We examined the expression of SSR and its correlation with recurrence and survival of patients. We discovered that SSR2 overexpression was negatively associated with survival of HCC patients from TCGA databases and the mutation of SSR2 was most among the four subunits of SSR protein. Additionally, in this study, we collected tumor and adjacent tissues from 125 cases of HCC patients. Through constructing tissue microarray, we have identified that SSR2 was highly expressed in HCC tumor tissues compared with adjacent normal tissues of hepatocellular carcinoma patients by immunohistochemistry assays. Furthermore, Kaplan-Meier survival analysis from our collected tissues revealed that the overexpression of SSR2 was inversely correlated with disease free survival and overall survival of HCC patients. We elucidated that SSR2 promotes proliferation, migration and invasion of HCC cells. SSR2 knockdown suppressed epithelial mesenchymal transition (EMT) of HCC cells.

**Conclusions:** These results collectively show that SSR2 is overexpressed in HCC tumor tissues, and it is an important factor in predicting survival of HCC patients. Additionally, it is involved in metastasis of HCC. These findings may help to exploit SSR2 as a novel factor in predicting prognosis and metastasis of HCC.

## Introduction

HCC is the fifth malignant cancer worldwide 1. Annual incidence of HCC in China is rising every year and accounts for the majority around the world 2-4. Despite various therapies that have been exploited to treat HCC, the 5-year survival rate is rather dismal 5-7. Metastasis was responsible for recurrence and caused most of death of HCC cases 8, 9. However, the molecular mechanisms underlying the metastasis and recurrence of HCC are far from clear. Exploiting novel targets for treating HCC and inhibiting HCC metastasis would help greatly to improve survival of HCC patients.

Epithelial-mesenchymal transition (EMT) is considered to be associated with metastasis and tumorigenesis 10, 11. EMT is characterized by the phenomenon that immobile epithelial cells become migratory mesenchymal cells 12, 13. EMT is modulated by various signaling pathways such as receptor tyrosine kinase, mitogen-activated protein kinase and so on 14, 15. Despite many new and exciting developments, several challenges remain to be addressed in order to understand more thoroughly the role of EMT in tumorigenesis and metastasis of malignant cancers.

Signal Sequence Receptor subunit 2 (SSR2), which is also known as translocon-associated protein subunit beta, is a glycosylated endoplasmic reticulum (ER) membrane receptor associated with protein translocation across the ER membrane 16, 17. SSR protein was considered to be involved in protein translation in eukaryotic cells 18. Importantly, SSR2 was reported to be involved in unfolded protein response on endoplasmic reticulum 19. There are few studies reporting the function of SSR2 in malignant cancer including HCC.

In this study, we discovered that SSR2 was upregulated in HCC tumor tissues. Our results revealed that SSR2 was strongly associated with encapsulation invasion, and tumor grade. Furthermore, SSR2 was associated with both overall survival and disease-free survival of HCC patients in both our tissues and other clinical cohorts. Taken together, SSR2 might be exploited as prognostic marker predicting survival. SSR2 was shown to promote proliferation of HCC cells. Moreover, SSR2 is critical for migration and invasion of HCC cells, in which EMT is involved, implying a pro-metastatic role of SSR2 in HCC. We unveiled a novel role of SSR2 in HCC tumorigenesis. These findings might help to elucidate its role in EMT-associated metastasis and recurrence in malignant cancers. It is of great significance to exploit SSR2 as a novel drug target for curbing HCC metastasis.

## Materials and methods

### Data sets

The mRNA expression of all SSR subunits in The Cancer Genome Atlas database (TCGA; https://tcgadata.nci.nih.gov/tcga/). The relationship of SSR and survival data were obtained from Gene Expression Profiling Interactive Analysis (GEPIA; http://gepia.cancer-pku.cn), and c-Bioportal databases were applied to analyze the mutation and expression of SSR2 in HCC patients from TCGA.

### Patient and tissue samples

Resected tumor tissues were collected from 125 HCC Patients from 2014-2016 in The Fifth Affiliated Hospital of Sun Yat-sen University. None of the patients has received any other chemotherapies, radiotherapy before surgery. Tissues were snap frozen in liquid nitrogen and stored at -80 °C for further analysis. Formalin-fixed, parrafin-embedded HCC tissues and adjacent non-tumor tissues were prepared from the 125 patients and subjected to further analysis. Clinical characteristics of all the patients were followed up and recorded. This study was approved by the Ethics Committee of The Fifth Affiliated Hospital of Sun Yat-sen University and conformed to the 1964 Declaration of Helsinki and its later amendments or comparable ethical standards. All tissues were collected after written informed consents were obtained from the patients.

### Cell lines and cell culture

Hep3b cells were obtained from Zhong Qiao Xin Zhou Biotechnology Co., Ltd. (Shanghai, China) and cultured in DMEM (Gibco, Big Cabin, UK) supplemented with 10% Fetal Bovine Serum (Gibco), supplemented with penicillin and streptomycin. Cells were cultured in a humidified incubator at 37 °C and 5% CO_2_.

### RNA isolation and quantitative PCR

Total RNA from cells and tissues were extracted using Total RNA Kit I (Omega, Norcross, USA). For RT-PCR, it was accomplished in TB Green Premix Ex Taq II (TaKaRa, Kusatsu, Japan) with QuantStudio 5 Flex Real-Time PCR (Thermo Fisher scientific, Waltham, USA). GAPDH was used as a reference gene.

### Cell proliferation analysis

Cell proliferation was measured using the IncuCyte Live-Cell Imaging System (Essen Bioscience, Ann Arbor, MI, USA). Hep3b cells (5,000 cells per well) were seeded in 96-well plate overnight to allow adherence and entry into the log-growth phase. The cells were placed in an incubator and imaged using a 10× objective. The IncuCyte Analyzer was used to collect real-time cellular confluence data based on acquired images. Cell proliferation is expressed as an increase in the percentage of confluence.

### Clonogenic assay

Briefly, cells were transfected with siRNAs for 24 hours. Subsequently, the cells were seeded in 6-well plates plated at with agarose at a density of 1000 cells per well. The cells were allowed to grow for approximately 14 days. Colonies were visualized with crystal violet staining (Sigma-Aldrich, Missouri, USA). Statistical results were obtained after comparing three independent experiments.

### Migration and invasion assays

Cells were seeded in trans-well chambers with 8-μm-pore inserts (Corning, NY, USA) for migration and invasion assays. For the migration assay, cells in serum-free medium were plated in uncoated inserts and incubated for 24 h. For the invasion assay, the inserts were coated with 100 μL of Matrigel (BD Biosciences, NJ, USA), and cells were plated in the serum-free medium described above for an incubation period of 24 hours. 500 µL culture medium containing 15% FBS was added to the lower chamber. The cells attached to the bottom of the membrane were fixed with 4% paraformaldehyde, then subjected to being stained with 5% crystal violet (Sigma-Aldrich), and counted at 200× magnification. The assays were repeated for three times.

### RNA interference and transfection

SSR2-specific siRNA or scramble siRNA (Genepharma, Shanghai, China) was transiently transfected with Hep3b cells with lipofectamine 2000 (Thermo Fisher Scientific, Waltham, USA), and the cells were subsequently cultured for 48 hours or the indicated periods. Total RNA was prepared and subjected to qPCR analysis or the total proteins were extracted for western blot analysis.

### Western blot analysis

Briefly, total cell lysates were extracted with RIPA buffer (Beyotime, Haimen, Jiangsu, China) containing a protease inhibitor cocktail (Thermo scientific, Waltham, USA) according to the manufacturer's instructions. The lysates were separated by denatured SDS-PAGE and transferred to nitrocellulose membranes. The membranes were incubated with the indicated antibodies. All antibodies were purchased from cell signaling technology (Beverly, MA, USA). Bands were visualized with ECL and imaged with ChemiDoc XRS (Bio-Rad, California, USA).

### Immunohistochemical (IHC) assays

Tissue slices were prepared from PFPE tissues. Briefly, the antigen retrieval was accomplished by citrate buffers. The slices were blocked with goat serum. Subsequently, the slides were incubated with primary antibodies overnight at 4 °C. Subsequently, a horseradish peroxidase-polymer anti-mouse/rabbit IHC kit (Zhongshan Goldbridge, Beijing, China) and incubated for 1 hour at room temperature. Then, the samples were developed with a diaminobenzidine (DAB) reagent, counterstained with hematoxylin and mounted with permount. The results were evaluated by two independent pathologists. Representative pictures were taken under the microscope under the guidance of pathologists. For tissue microarray, all tissues with the diameter 1.5 mm were obtained and aligned in a prepared array. Slices were prepared and followed by the IHC protocol.

### Statistical analysis

Data are presented with means ± standard deviation (SD). Statistical analysis was conducted using GraphPad Prism 7. Significant differences were evaluated by using Student's t-*test*. The χ^2^ test was used to analyze the relationships between categorical variables and p<0.05 was considered statistically significant. Kaplan-Meier survival and the log-rank tests were used to analyze the correlation of SSR2 expression and HCC patients' survival.

## Results

### SSR2 was upregulated in HCC tumor tissues and correlates with patients' survival in public cohorts

**Figure 1:**
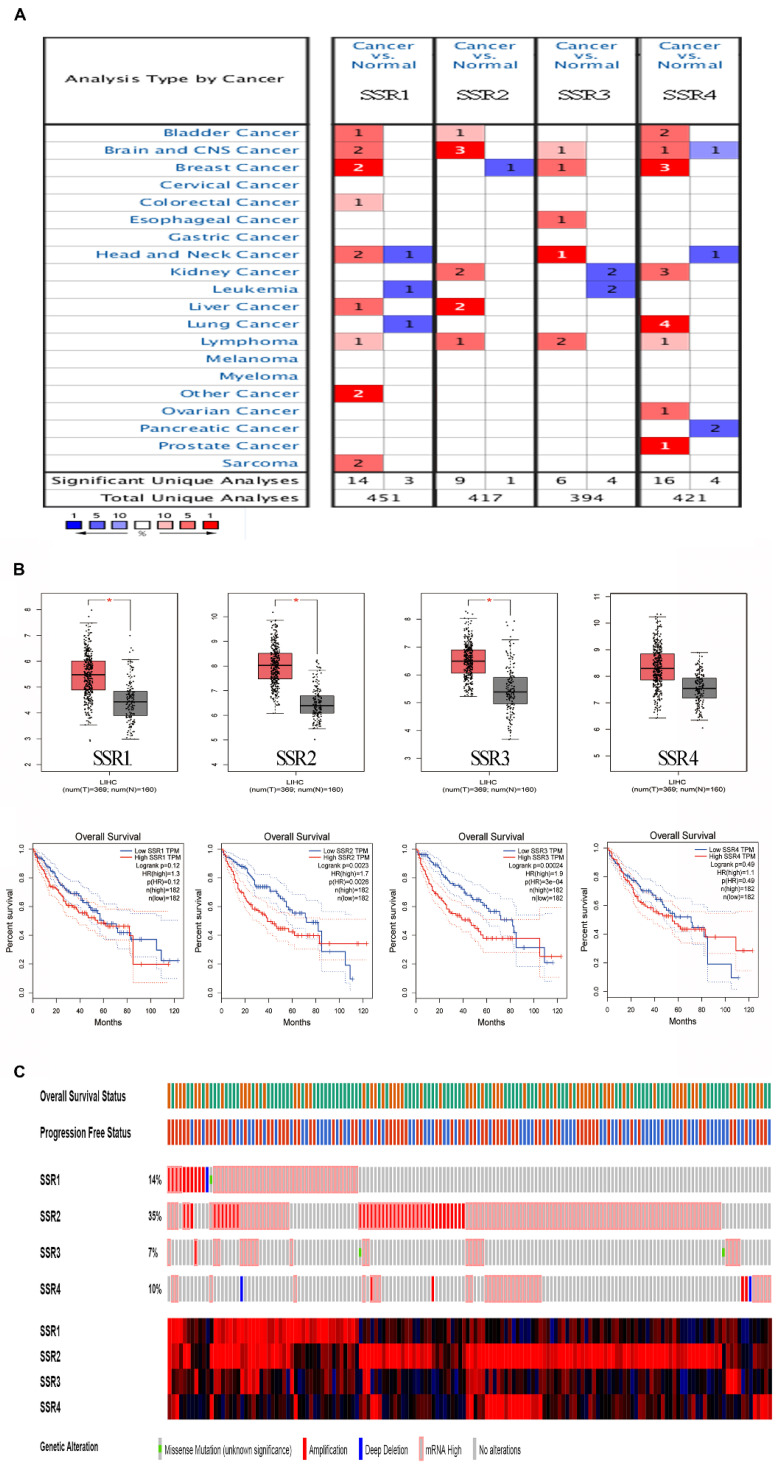
** The expression and survival analysis of SSR subunits in various cancers (Public cohorts).** Expression of SSR subunits in tumor and normal tissues in various cancer. Upper: Expression of SSR subunits in HCC; Lower: Survival analysis of SSR subunits in HCC patients. The mutation and expression of SSR subunits in HCC patients were analyzed from TCGA sequencing data.

To make clear the role of SSR2 in tumor progression, we tentatively examined the expression of SSR in various cancers. As shown in Figure 1A, we discovered that four subunits of SSR shown upregulation in plethora of malignant tumors. Then we evaluated the correlation of SSR subunits expression and survival of HCC patients. From Figure 1B, we found the expression of SSR2 and SSR3 were inversely correlated with survival of HCC patients. Moreover, we detected the expression pattern of SSR in c-bioportal database. As shown in Figure 1C, among four subunits of SSR protein, the mutation and expression of SSR2 in HCC were most evident. Above results from public database led us to further validate the expression and clinical significance of SSR2 in our own obtained HCC tissues.

### SSR2 was overexpressed in HCC tissues and correlated with poor prognosis of HCC patients

**Figure 2:**
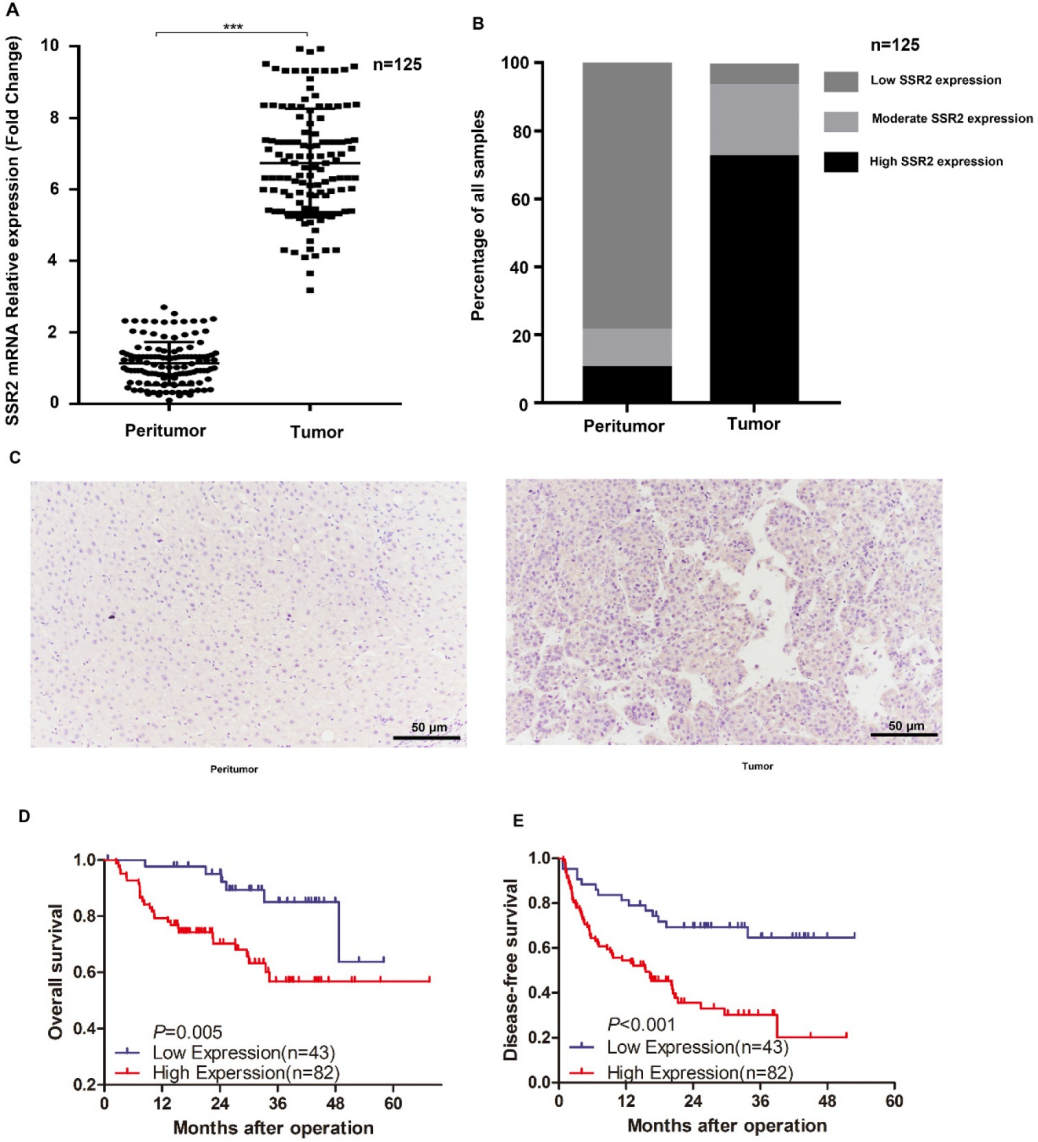
** SSR2 expression in our own collected HCC tumor tissues and their counterparts.** The mRNA expression of SSR2 in HCC tumor and peritumor tissues. *** indicates p<0.05. Statistical analysis of IHC results from tissue microarray. The percentage of samples with differential expression of SSR2 was shown in both tumor and peritumor parts. IHC analysis of the expression of SSR2 in HCC tumor and peritumor tissues. D-E: Survival analysis delineating the relationship of SSR2 expression and overall survival or disease-free survival.

Importantly, in our previously collected tumor tissues and corresponding non-tumor tissues, we discovered that SSR2 mRNA expression was markedly elevated in tumor tissues (Figure 2A). Subsequently, we examined the expression of SSR2 by IHC assays. We discovered SSR2 expression was elevated compared with the corresponding adjacent normal tissues (Figure 2B), From our tissue microarray, we found that high SSR2 expression accounts for about 65.6%, while most of adjacent non-tumor tissues, SSR2 expression was quite low (Figure 2C). Survival analysis indicated that SSR2 was negatively correlated with both overall survival and disease-free survival of HCC patients (Figure 2D-E). These results collectively show that SSR2 was upregulated in HCC tumor tissues and was clinically significant.

### SSR2 was an independent risk factor for prognosis of HCC

Our results have indicated that SSR2 overexpression in tumor tissues was associated with survival of HCC patients. To determine the relationship of SSR2 expression and clinicopathological characteristics, we analyzed the relationship of SSR2 expression and the clinical parameters (Table 1), high expression of SSR2 was positively correlated with encapsulation invasion (p=0.01) and tumor grade (p=0.03). Moreover, a multivariate Cox regression analysis of risk factors associated with overall survival revealed that high SSR2 expression (HR, 3.18; 95% CI, 1.05-9.68; p=0.04) and AFP>200ng/mL (HR, 3.37;95%CI, 1.33-8.52; p=0.01) were independent prognostic factors for overall survival (Table 2). As shown in Table 3, a multivariate Cox regression analysis of risk factors associated with disease-free survival revealed that the high expression of SSR2 (HR, 3.32; 95% CI, 1.44-7.65; p=0.01) and late stage (HR, 3.05; 95% CI, 1.10-8.51; p=0.03) were independent prognostic factors for disease-free survival. To examine the accuracy of predicting overall survival with SSR2 and serum AFP levels, ROCs were used. The results (Table 4) demonstrate that combined SSR2 and AFP were more sensitive compared with AFP alone in predicting survival in HCC patients.

### SSR2 has a tumorigenic role in HCC

**Figure 3:**
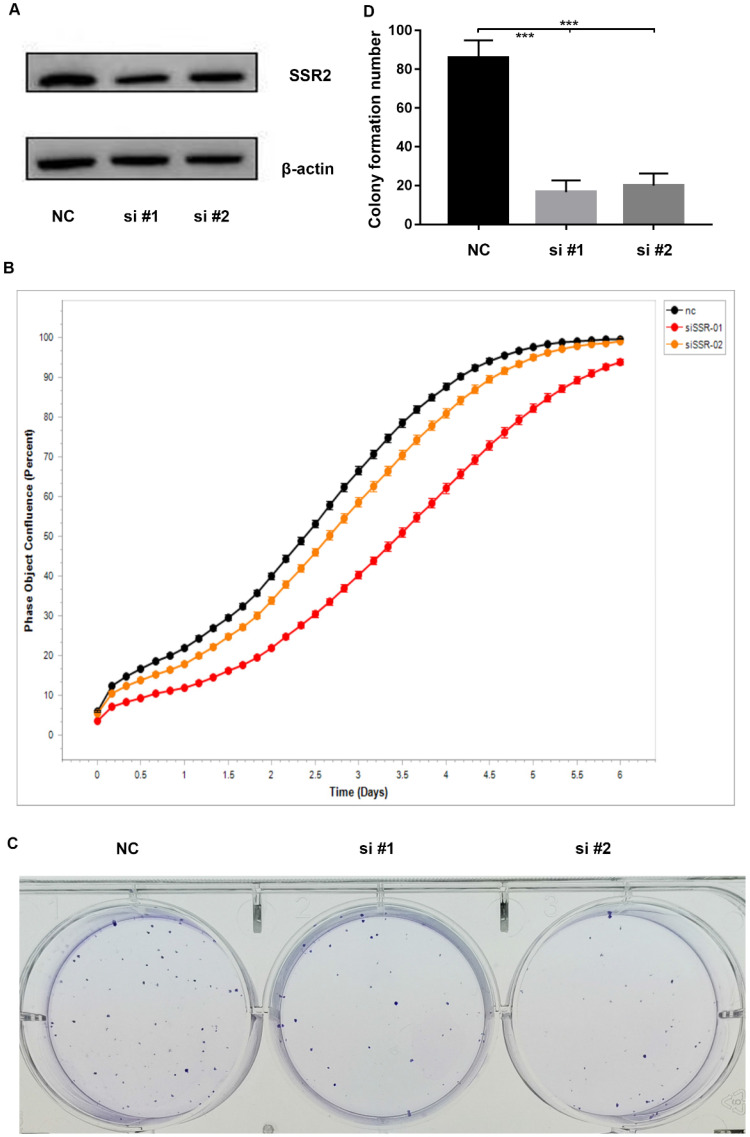
** SSR2 has a pro-tumorigenic role in HCC cells.** SSR2 was successfully knocked down validated by western blot assays. β-actin was used as a loading control. NC was transfected with scramble siRNA, while si#1 and si#2 were transfected with siRNA targeting SSR2. The proliferation of control (NC) and SSR2 knockdown cells by IncuCyte assays. Colony formation analysis of the cells treated with scramble siRNA or SSR2 specific siRNA. Statistical analysis of colony formation results. The difference between NC and interference group was statistically significant as *** (p<0.005).

To investigate the role of SSR2 in HCC tumorigenesis, we performed a series of experiments. Firstly, we knocked down the expression of SSR2 by using siRNA transfection into cells. From Figure 3A, we vividly show that SSR2 was successfully knocked down as demonstrated in western blot results. Then we detected the proliferation of Hep3b cells in case of SSR2 knockdown. We discovered that the proliferation of Hep3b cells was inhibited in case of SSR2 knockdown (Figure 3B). Furthermore, we carried out colony forming assay to make clear the role of SSR2 in HCC tumorigenesis. As you can see from Figure 3C, the colony number formed decreased with the knockdown of SSR2 compared with control cells. Statistical analysis of the colony formation assays shown that SSR2 knockdown caused statistical decrease of colony formation ability of Hep3b cells (Figure 3D).

### SSR2 play a pivotal role in migration and invasion of HCC cells

**Figure 4:**
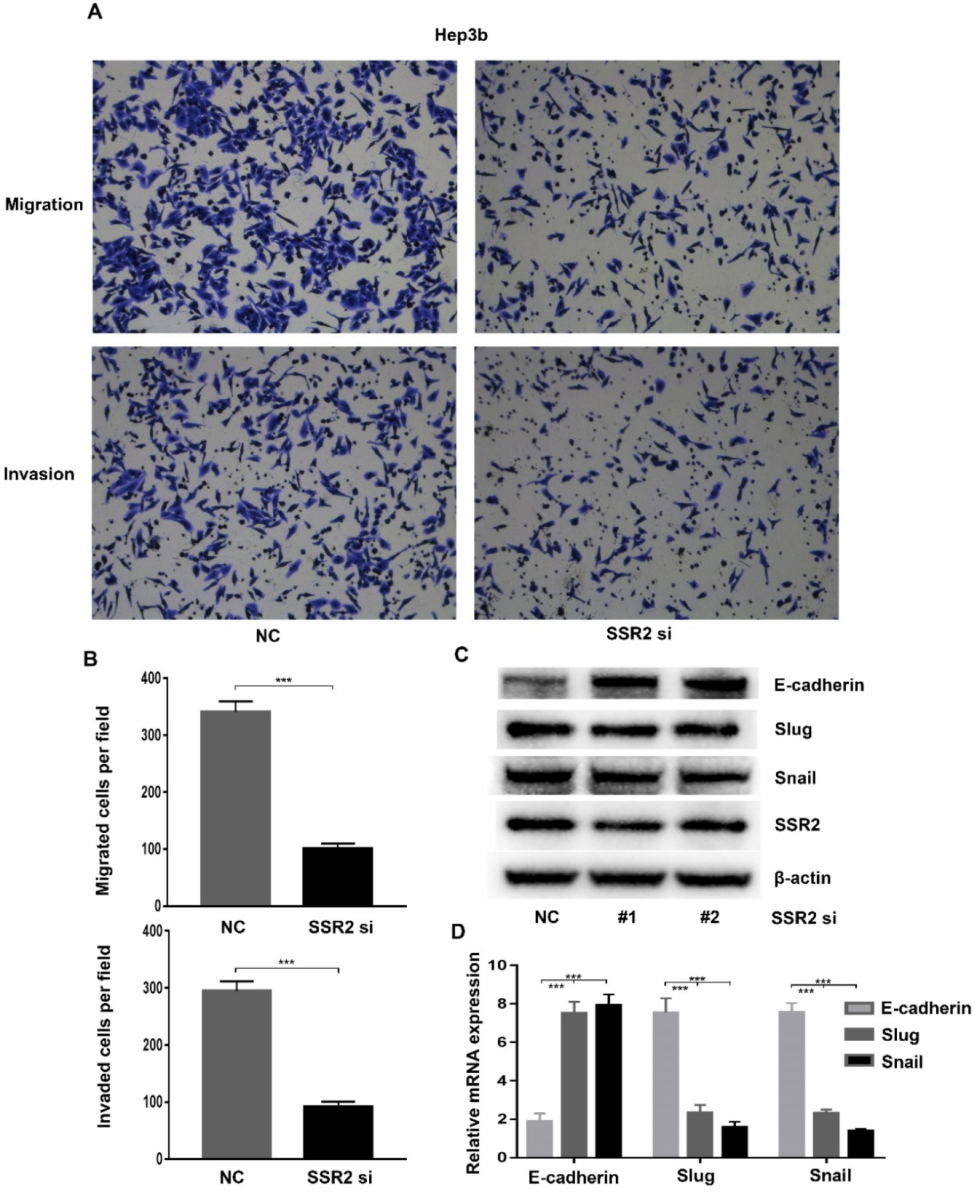
** SSR2 promotes migration and invasion through modulating EMT in HCC cells.** The migration and invasion of Hep3b cells were analyzed by trans-well assays. Representative pictures were shown in both NC and siRNA group. (Upper: migration; Lower: invasion). Statistical analysis results were shown from migration and invasion assays. Five random fields were picked up and the differences were statistically significant (*** indicates p<0.005). Western blot assays detecting the expression of EMT-related markers in HCC cells. qPCR analysis of EMT-related molecules E-cadherin, Slug and Snail after SSR2 knockdown.

Metastasis was the predominant reason of death of HCC patients. To further clarify the role of SSR2 in HCC metastasis. We investigated the role of SSR2 in migration and invasion of HCC cells. We attenuated the expression of SSR2 using siRNA transfection. Western blot was applied to validate the knockdown efficiency of SSR2. By using trans-well assays, we discovered that, compared with control group, the migration and invasion of Hep3b cells decreased greatly (Figure 4A). Statistical analysis revealed that SSR2 knockdown vividly suppressed the migration and invasion of Hep3b cells (Figure 4B). The migration and invasion were essential steps for metastasis; these results prompted us to investigate the role of SSR2 knockdown on EMT. Importantly, SSR2 knockdown enhanced the expression of E-cadherin, suppressed the expression of snail of slug as shown from western blot results and qPCR assays respectively (Figure 4C-D). Those results indicated that SSR2 knockdown modulated the EMT in HCC cells, which might be the mechanism underlying the pro-tumorigenic and pro-metastasis role of SSR2.

## Discussion

SSR2 was previously reported as an ER-associated protein 20, 21. There are few studies reporting the role of SSR2 in HCC. Herein this study, we unveiled a novel pro-metastasis role of SSR2 in HCC, pointing out the tumorigenic and pro-metastatic role of SSR2. These results were of great clinical significance, since we observed the overexpression of SSR2 was inversely associated with survival of HCC patients. All of our results indicate that SSR2 might be exploited as a biomarker for prognosis and predicting relapse of HCC patients.

SSR2 have been reported to be involved in unfolded protein response in eukaryotic cells 22. SSR3, a subunit of SSR protein was shown to be dysregulated in malignant cancers 23. But little is known about the role of SSR2 in HCC. Therefore, for the first time, we proposed that SSR2 might play a pivotal role in HCC. Data mining in TCGA and GEPIA databases results supported our hypothesis that SSR2 was overexpressed in HCC tumor tissues. Also, we validated the expression of SSR2 in our previously collected resected HCC tissues. Consistently, SSR2 expression was elevated in tumor tissues compared with adjacent normal tissues. Kaplan-Meier survival analysis also validated the conclusion that SSR2 overexpression implies a poor prognosis. Additionally, SSR2 was validated to be independent prognostic factor for overall survival and disease-free survival. Critically, SSR2 have been shown to be involved in proliferation, migration and invasion of HCC cells. These findings convincingly elucidate the clinical significance and biological function of SSR2 in HCC. Targeting SSR2 might be feasible for curbing the progression and metastasis of HCC.

Plenty of reports show that EMT was an essential and critical step for metastasis of malignant cancer 11, 24, 25. EMT-associated factors including E-cadherin, Snail, Slug were diversely involved in signaling pathway modulating metastasis 26-28. Elucidation EMT-associated signaling network would add another layer of evidence to disclosing mechanisms accounting for metastasis and recurrence of HCC 29, 30. Elucidating the role of SSR2 in modulating EMT and its associated molecules would unveil the role and mechanism of SSR2 in HCC tumorigenesis and metastasis. Herein this study, we discovered that SSR2 inhibition attenuated EMT occurrence accompanied by upregulation of E-cadherin, decreased expression of Slug and Snail. Those results would help to make clear how SSR2 links to metastasis of HCC, providing novel proof for linking UPR with metastasis.

However, additional experiments are required to delineate the detailed molecular mechanisms underlying how SSR2 was upregulated in HCC tumor tissues and how SSR2 promotes proliferation, migration and invasion of HCC cells. How EMT was modulated by SSR2, directly or indirectly? Whether SSR2 has regulation on other subunits of SSR protein also warrants further study.

## Figures and Tables

**Table 1:** Correlation between SSR2 expression and clinicopathological characteristics of hepatocellular carcinoma

Variables	n	SSR2 expression		p Value
		Low (43)	High (82)	
**Gender**				
Male	109	37	72	0.78
Female	16	6	10	
**Age (years)**				
<50	58	23	35	0.26
≥50	67	20	47	
**AFP (ng/mL)**				
<200	91	32	59	0.84
≥200	34	11	23	
**Size (cm)**				
<5	79	26	53	0.70
≥5	46	17	29	
**Tumor nodule number**				
<2	106	40	66	0.07
≥2	19	3	16	
**Cirrhosis**				
Negative	67	25	42	0.57
Positive	58	18	40	
**MVI**				
Negative	70	26	44	0.57
Positive	55	17	38	
**Stage**				
Early (I&II)	96	35	61	0.50
Late (III&IV)	29	8	21	
**Encapsulation invasion**				
Negative	80	34	46	0.01
Positive	45	9	36	
**Grade**				
Well	107	41	66	0.03
Poor	18	2	16	

**Table 2:** Univariate and multivariate Cox regression analyses of risk factors associated with overall survival in HCC patients

Variables	Univariate analysis			Multivariate analysis		
	HR	95%CI	p value	HR	95%CI	p value
**SSR2 expression**	3.20	1.21-8.49	0.02	3.18	1.05-9.68	0.04
(High vs. Low)						
**Gender**	1.25	0.40-3.92	0.70			
(Male vs. Female)						
**Age (years)**	0.50	0.23-1.12	0.09			
(≥50 vs.<50)						
**AFP (ng/mL)**	3.61	1.54-8.42	<0.01	3.37	1.33-8.52	0.01
(≥200 vs. <200)						
**Size (cm)**	2.53	1.13-5.67	0.02	1.68	0.65-4.37	0.29
(≥5 vs. <5 )						
**Tumor nodule number**	0.95	0.31-2.87	0.93			
(≥2 vs.<2)						
**Cirrhosis**	1.28	0.58-2.81	0.55			
(Positive vs. Negative)						
**MVI**	2.29	1.02-5.10	0.04	1.82	0.74-4.45	0.19
(Positive vs. Negative)						
**Stage**	4.34	1.79-10.52	<0.01	2.73	0.98-7.41	0.05
(Late vs. Early)						
**Encapsulation invasion**	2.25	1.01-5.04	<0.05	1.29	0.49-3.41	0.61
(Positive vs. Negative)						
**Grade**	1.89	0.66-5.35	0.23			
(Poor vs. Well)						

**Table 3:** Univariate and multivariate Cox regression analyses of risk factors associated with disease-free survival in HCC patients

Variables	Univariate analysis			Multivariate analysis		
	HR	95%CI	p value	HR	95%CI	p value
**SSR2 expression**	3.59	1.65-7.83	0.02	3.32	1.44-7.65	0.01
(High vs. Low)						
**Gender**	0.88	0.31-2.51	0.81			
(Male vs. Female)						
**Age (years)**	0.65	0.32-1.31	0.23			
(≥50 vs.<50)						
**AFP (ng/mL)**	1.01	0.46-2.22	0.99			
(≥200 vs. <200)						
**Size (cm)**	1.68	0.80-3.51	0.17			
(≥5 vs. <5 )						
**Tumor nodule number**	0.77	0.29-2.06	0.61			
(≥2 vs.<2)						
**Cirrhosis**	1.66	0.82-3.39	0.16			
(Positive vs. Negative)						
**MVI**	2.53	1.22-5.24	0.01	2.20	1.00-4.84	0.05
(Positive vs. Negative)						
**Stage**	3.71	1.45-9.51	0.01	3.05	1.10-8.51	0.03
(Late vs. Early)						
**Encapsulation invasion**	2.44	1.14-5.23	0.02	1.49	0.64-3.47	0.36
(Positive vs. Negative)						
**Grade**	1.14	0.42-3.11	0.80			
(Poor vs. Well)						

**Table 4:**
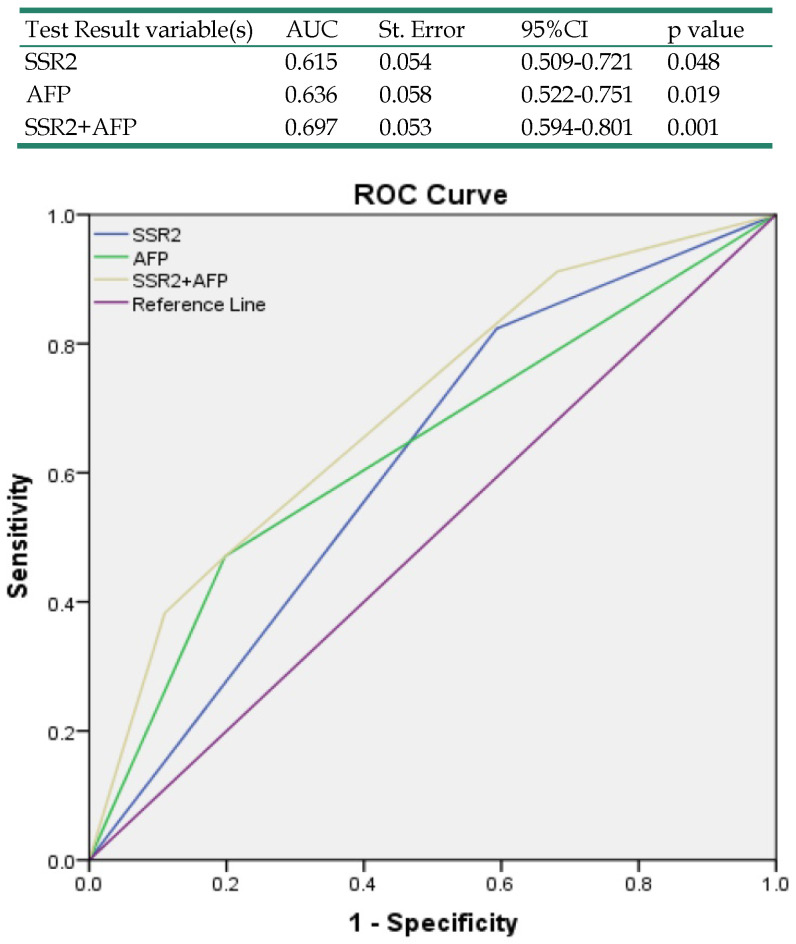
Receiver operating characteristics curve analysis of survival according to variables

## Abbreviations

EMT): Hepatocellular carcinoma (HCC
